# Preparation of a Lignin-Based Cationic Flocculant and Its Application in Kaolin Suspension Treatment

**DOI:** 10.3390/polym16081131

**Published:** 2024-04-17

**Authors:** Yan Li, Suling Yao, Xianshu Dong, Yuping Fan, Xiaomin Ma, Benkang Zhu, Ming Chang

**Affiliations:** 1 College of Mining Engineering, Taiyuan University of Technology, Taiyuan 030024, China; 2 State Key Laboratory of Mineral Processing, Beijing 100160, China

**Keywords:** lignin, grafted cationic flocculant, solid-liquid separation, FBRM, kaolin

## Abstract

The preparation of an environmentally friendly and efficient flocculant for solid-liquid separation in industrial wastewater is highly important. In this study, a novel cationic flocculant (AL-g-PAMA) was synthesized by a thermal initiation method using alkali lignin (AL) as the main chain and acrylamide (AM) and methacrylamido propyl trimethyl ammonium chloride (MAPTAC) as the grafted side chains. The structure, thermal stability, and surface morphology of the copolymers were investigated by various characterization methods. The results indicated the successful synthesis of AL-g-PAMA. AL-g-PAMA was applied to improve solid-liquid separation in kaolin suspensions. The results showed that AL-g-PAMA had excellent flocculation-sedimentation and dewatering efficiency. When the dosage of AL-g-PAMA #5 was 600.0 g/t_(s)_, the thickness of the compressed layer was 2.2 cm, the floc settling velocity was 24.1 cm/min, and the transmittance of the supernatant was 84.0%. The moisture content of the filter cake decreased from 55.0% to 43.4% after treatment with AL-g-PAMA #5. The results of zeta potential and focused beam reflectance measurement (FBRM) analysis indicated that bridging and electroneutralization were the main flocculation mechanisms. Therefore, this study extends the potential for using lignin as a bioflocculant and provides a feasible approach to efficiently purify high-turbidity wastewater.

## 1. Introduction

Significant quantities of clay minerals are present in wastewater discharged from industrial activities, such as mineral processing, papermaking, construction, and oil exploitation [1]. Kaolin is a major component of such clay minerals [2]. Fine kaolin particles are subject to hydration, selective adsorption, dissolution, and lattice replacement in sludge water, resulting in negatively charged particle surfaces. The surface of particles forms a hydration film and an electric double layer, which results in strong hydration repulsion and steric hindrance effects, making precipitation and dewatering increasingly difficult [3]. Moreover, fine kaolin particles have difficulty overcoming various forms of resistance and disturbance when driven by gravitational forces, which can lead to settling problems [4]. Therefore, separating clay minerals from industrial wastewater is of great theoretical and practical importance [1,5].

The use of flocculants to treat dispersed industrial wastewater has been demonstrated to be effective in improving the efficiency of solid-liquid separation [6]. In recent years, natural flocculants have become a green solution for treating a wide variety of industrial wastewater due to their wide range of sources and biodegradability [7]. Lignin is the most abundant renewable aromatic polymer in nature [8]. It provides a good source of reactants for the production of value-added products because it is inexpensive and readily available [9]. However, lignin is rarely utilized as a value-added product [10]. For example, most lignin separated from wood pulp waste streams is burned as fuel or disposed of as waste [10], which leads to the loss of valuable natural polymers and causes an increase in greenhouse generation. Therefore, the development of value-added functional materials from lignin is highly desirable [11].

Lignin maintains its stable structure in water due to its rigid three-dimensional skeletal framework, which can significantly improve its capacity to flocculate through the sweep effect [12]. Lignin features phenolic hydroxyl and carboxyl groups, which endow it with a strong potential for modification and enable it to be modified and used as a flocculant [13]. Most previous studies have focused on the Mannich reaction of lignin. The positively charged amino groups introduced by the Mannich reaction can neutralize the charge on negatively charged micellar particles in wastewater [14]. Wang et al. [12] prepared a polymer with highly efficient flocculation properties using lignin and acrylamide (AM) as raw materials in a UV-induced aqueous-phase copolymerization system via the Mannich reaction, which provided an innovative and green strategy for the development of novel lignin-based flocculants. Sheehan et al. [15] synthesized a biodegradable water-soluble flocculant (WSP) by grafting hydrophilic L-lysine onto the aromatic backbone of depolymerized lignin via the Mannich reaction. The test results showed that a 10–20 mg/L lignin-based WSP could reduce the turbidity of kaolin suspensions by more than 95%. However, Mannich reaction-modified lignin has disadvantages, such as low reactivity and low molecular weight, which limit its application as a flocculant [16]. In previous studies, the number of Mannich active sites on lignin has typically been increased using phenolic pretreatment [10]. Wang et al. [17] increased the number of Mannich active sites of lignin by phenolization pretreatment and then conducted sequential grafting of amino and sulfonate functional groups onto the phenolized lignin in an alkaline solution via the Mannich reaction and sulfomethylation, respectively, to synthesize a lignin-based adsorbent. The use of phenol solutions and concentrated sulfuric acid in the phenolization process and the use of polluting solvents in the Mannich reaction prompt environmental and economic concerns. These chemical processes can lead to the generation of effluents and wastes that pollute the environment and can entail expensive waste disposal and environmental remediation costs [12].

Graft polymerization of lignin is conducted through a free radical polymerization reaction, whereas the Mannich reaction occurs through polycondensation. Free radical polymerization reactions are more likely to result in the formation of reactive intermediates, thus enhancing the reactivity of the system. In recent years, graft copolymerization has been frequently used to prepare various environmentally friendly lignin-based flocculants with improved flocculation performance. Moore et al. [18] synthesized a cationic hydrolyzed lignin polymer using free-radical polymerization with [2-(methacryloyloxy)ethyl] trimethylammonium chloride (METAC) and hydrolysis lignin (HL) and then examined the flocculation efficiency of the lignin-based flocculants in simulated wastewater. Their results indicated that HL-METAC could remove up to 60% of the TOC present. In addition to the graft polymerization of binary compounds described above, ternary compounds and multicomponent compounds can be synthesized by graft polymerization [19,20]. Chen et al. [21] synthesized a lignin-grafted cationic flocculant (L-CPA) via a “grafting to” strategy using acrylamide (AM), methylacryloyloxyethyltrimethyl ammonium chloride (DMC), and enzymatically hydrolyzed lignin (EHL) as the raw materials. Their results showed that the resulting flocculant had a favorable flocculation effect. When the dosage of L-CPA was 4 mg/L, the maximum light transmittance of the kaolin suspension was 82%. Therefore, grafting functional monomers onto lignin is a feasible approach for improving the reactivity of lignin.

Kaolin particles are extremely fine and have negative surface charges; therefore, they do not easily settle in water and are prone to sludge formation. Flocculation is one of the most economical and effective methods for settling clay minerals in industrial processes. Lignin has many functional groups, and lignin modification for use as a flocculant is a promising treatment approach. In this study, to address the issue of settling kaolin, the molecular weight of lignin was increased by the introduction of AM to enhance bridging during flocculation, and the positive charge number and space volume of lignin were increased by the introduction of MAPTAC to further enhance the adsorption–electroneutralization and net trapping–scavenging effects of lignin during flocculation. This increase in molecular weight and the number of positive charges was designed to enhance the interaction of lignin with particles during the flocculation process. To test our hypothesis, we explored the flocculation and dewatering performance of AL-g-PAMA on kaolin suspensions. This research provides a new approach to utilizing natural polymers for cost-effective water treatment.

## 2. Materials and Methods

### 2.1. Materials

**Figure 1:**
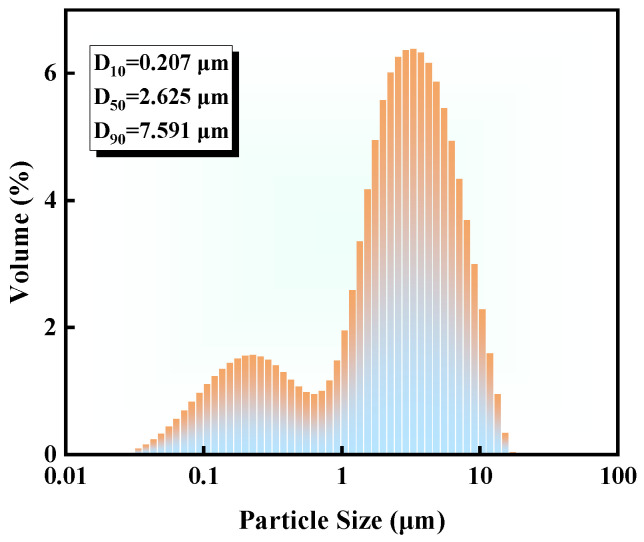
Particle size distribution of kaolin samples.

Alkaline lignin (AL) was purchased from Beijing Huamaike Biotechnology Co., Ltd. (Beijing, China). AM and absolute ethanol were supplied by Tianjin Tianli Chemical Reagent Co., Ltd. (Tianjin, China). Cationic polyacrylamide (CPAM) was purchased from Shanghai Aladdin Bio-Chem Technology Co., Ltd. (Shanghai, China). Methacrylamido propyl trimethyl ammonium chloride (MAPTAC, 50% in H_2_O) was purchased from Shanghai Macklin Biochemical Technology Co., Ltd. (Shanghai, China). Potassium persulfate (KPS) was purchased from Sinopharm Chemical Reagent Co., Ltd. (Shanghai, China). All the solutions used in the experiments were prepared with deionized water. Kaolin (average particle size 3.4 μm) was obtained from Tianjin Hengxing Chemical Reagent Manufacturing Co., Ltd. (Tianjin, China). A laser particle size analyzer (S3500, Microtrac MRB, Montgomeryville, PA, USA) was used to analyze the particle sizes of the kaolin samples, as shown in Figure 1.

### 2.2. Preparation of AL-g-PAMA

**Scheme 1:**
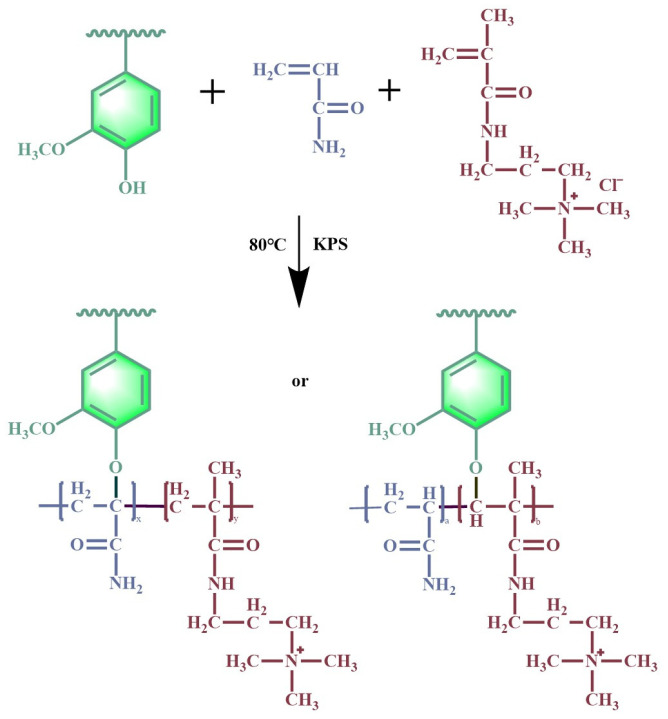
Free radical reaction of the grafted polymer (AL-g-PAMA).

As shown in Scheme 1, AL was mixed with AM and MAPTAC in a three-neck flask, and then deionized water was added. Subsequently, the oxygen in the reaction vessel was removed by N_2_ (99.0%) sparging at room temperature (approximately 25 °C) for 30 min. Then, KPS was added as an initiator, and the reaction was allowed to continue for 2 h at 80 °C in a water bath. After 2 h, air was introduced to terminate the reaction. After the solution was left undisturbed at room temperature for 2 h, a clear gel-like solid product formed. Then, the obtained gel product was purified using excess absolute ethanol. The product gradually transformed from a clear gel to a brownish-yellow precipitate. The resulting polymer was continuously dried in a vacuum oven at 50 °C to a constant weight. Finally, the obtained product was ground to a powder using a mortar and pestle for subsequent characterization and application.

### 2.3. Characterization of AL-g-PAMA

The functional group structures of the samples were characterized by FTIR (Nicolet iS20, Thermo, Waltham, MA, USA). The resolution was 4 cm^−1^, the number of scans was 32, and the range of wavenumbers tested was 600–4000 cm^−1^. The molecular structures of the samples were characterized by ^1^H NMR (AVANCE Model DRX-500, Bruker, Karlsruhe, Germany), and the ^1^H NMR spectra of the samples were obtained by dissolving the samples in D_2_O. The structures of the samples were characterized by XRD (MiniFlex 600 model, Rigaku, Tokyo, Japan) with a scanning range of 5–85° and a scanning speed of 8°/min. After the samples were sprayed with gold using a Quorum SC7620 sputter ion coater, their surface morphology was determined by SEM (MIRA LMS, TESCAN, Brno, Czech Republic). The thermal decomposition properties of the polymers were determined by a thermogravimetric analyzer(SDT-650, TA, Newcastle, DE, USA). The temperature was increased from room temperature to 700 °C at a heating rate of 10 °C/min and a nitrogen flow rate of 100 mL/min.

### 2.4. Grafting Efficiency of PAMA and Intrinsic Viscosity Determination

#### 2.4.1. Determination of Grafting Efficiency

The grafting efficiency of PAMA (GE) was determined via gravimetric methods and calculated as follows [22]:

(1) $$GE=\frac{M_{3}-M_{0}}{M_{0}+M_{1}+M_{2}}\times 100\%$$

where $M_{0\;}$ is the mass of AL, g; $M_{1}$ is the mass of AM, g; $M_{2}$ is the mass of MAPTAC, g; and $M_{3}$ is the total mass of the graft copolymer after purification, g.

#### 2.4.2. Determination of Intrinsic Viscosity

The intrinsic viscosity of the polymers (η) was determined via the one-point method [23]. Measurements of the intrinsic viscosity of the polymer solutions were conducted using a Ubbelohde viscometer (capillary diameter of 0.46 mm) at a constant temperature of 30 °C. First, 0.08–0.1 g of polymer was accurately weighed into a 200 mL volumetric flask. After adding approximately 90 mL of deionized water, the volumetric flask containing the sample was placed in a thermostatic shaker at 30 ± 0.05 °C and shaken to fully dissolve the polymer. Then, 100 mL of 2 mol/L NaCl solution was added to the volumetric flask. After sufficient shaking, the sample solution in the volumetric flask was diluted to scale with deionized water. Finally, the sample test solution was filtered through a sand core funnel, and the intrinsic viscosity was determined by the one-point method. Equation (2) was used to calculate the intrinsic viscosity of the polymer as follows:

(2) $$\left[\eta \right]=\frac{\sqrt{\frac{2\;-\;\ln3}{\eta_{r}\;-\;\ln\eta_{r}\;-\;1}\left(\eta_{r}\;-\;1\right)-2\sqrt{\frac{\eta_{r}\;-\;\ln\eta_{r}\;-\;1}{2\;-\;\ln3}}}}{c(\sqrt{\frac{2\;-\;\ln3}{\eta_{r}\;-\;\ln\eta_{r}\;-\;1}}-1)}$$

where η is the intrinsic viscosity of the polymer, dL/g; c  is the concentration of the polymer solution, g/L; and $\eta_{r}$ is the relative viscosity (dL/g) of the polymer, as calculated by Equation (3) as follows:

(3) $$\eta_{r}=\frac{t}{t_{0}}$$

where t and $t_{0}$ are the time that the polymer solution and the 1 mol/L NaCl solution spent flowing through the upper and lower scale lines of the Ubbelohde viscometer, respectively.

### 2.5. Flocculation-Sedimentation and Dewatering Experiments

#### 2.5.1. Flocculation and Sedimentation Experiments

First, 20 g of kaolin was added to 1 L of deionized water and then stirred evenly at 500 rpm. The prepared suspension was used for flocculation (pH = 7). Subsequently, an appropriate volume of the prepared flocculant solution was injected into a 250 mL settling tube containing a kaolin suspension at a concentration of 20 g/L. Next, the tube was capped and inverted five times at room temperature. Then, the settling tube was quickly placed upright on the test bench, and the level of the clarified liquid was recorded over time as the suspended particles settled. After 5 min, the height of the accumulated flocs was recorded. The transmittance and absorbance of the supernatant were determined with a Shanghai Jinghua 721 visible spectrophotometer. The zeta potential of the supernatant was measured by a zeta potential analyzer (PANalytical, Malvern Instruments, Malvern, UK).

#### 2.5.2. Dewatering Experiments

After the suspension settled, a vacuum pump was turned on, the pressure was set to 0.08 MPa, and the suspension was poured into a Brinell funnel for vacuum filtration. After filtration, the dewatering time was recorded. The filter cake was dried at 105 °C for 2 h. The mass of the filter cake before and after drying was recorded. The moisture content of the filter cake was calculated according to Equation (4) as follows:

(4) $$Moisture\;content\%=\frac{M_{1}-M_{2}}{M_{1}-M_{0}}\times 100\%$$

where $M_{0}$ is the total mass of the beaker and filter paper, g; $M_{1}$ is the total mass of the beaker, filter cake, and filter paper after filtration, g; and $M_{2}$ is the total mass of the beaker, filter cake, and filter paper after drying, g.

The dewatering rate was calculated according to Equation (5) [24] as follows:

(5) $$u=\frac{V}{T\cdot \pi \cdot R^{2}}$$

where u is the dewatering rate, cm/min; V is the volume of filtrate, mL; T is the dewatering time, s; and R is the radius of the Brinell funnel, cm.

### 2.6. FBRM Test

The flocculation behavior and properties of the flocs formed in kaolin suspensions were monitored in real time using an FBRM instrument (G400, Mettler Toledo, Zurich, Switzerland). Kaolin (5 g) was added to 250 mL of deionized water and mixed at 300 rpm for 1 day to ensure that the particles were completely dispersed. Tests were performed at 150 rpm to keep the particles in suspension, and the flocculant was added to this suspension 2 min after the beginning of the FBRM test. Recordings were made every 2 s to dynamically monitor the solution after the addition of the flocculant.

## 3. Results and Discussion

### 3.1. Structural Characteristics of AL-g-PAMA

#### 3.1.1. FTIR Analysis

**Figure 2:**
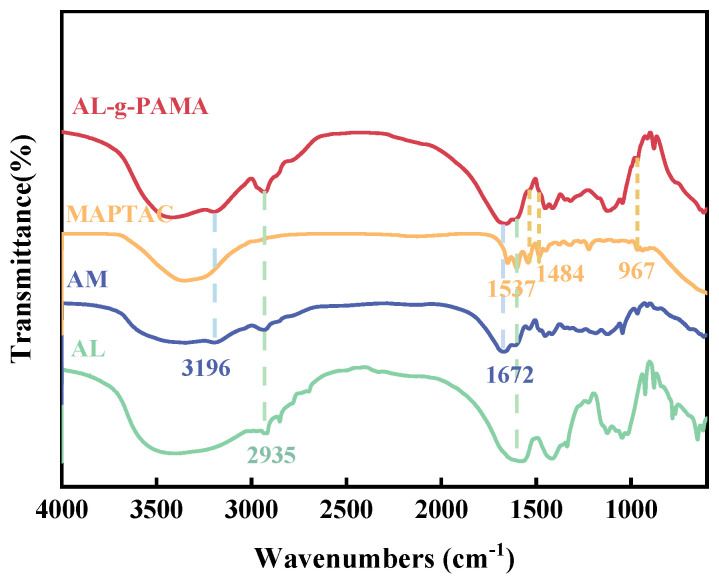
FTIR spectra of AL, AM, MAPTAC, and AL-g-PAMA.

Figure 2 shows the FTIR spectra of AL, AM, MAPTAC, and AL-g-PAMA. Compared to the FTIR spectrum of AL, that of AL-g-PAMA showed an amino group (N-H) stretching vibrational peak at 3196 cm^−1^ that was not present before grafting; this amino group was attributed to the introduction of the amide group (–NH_2_) in AM [25]. The absorption peak at 2935 cm^−1^ corresponded to the asymmetric stretching vibration of the hypomethyl group (–CH_2_) [26]. The telescoping vibrational peak located at 1672 cm^−1^ was attributed to the introduction of carbonyl (C=O) in AM and MAPTAC [27]. The characteristic absorption peaks at 1454 and 967 cm^−1^ corresponded to the bending vibration of –CH_2_ adjacent to the quaternary ammonium and the telescoping vibration of (–N^+^(CH_3_)_3_) in MAPTAC, respectively. In addition, in contrast to the FTIR spectra of AM and MAPTAC, that of AL-g-PAMA retained the telescopic vibrational peak of the AL phenyl ring at 1600 cm^−1^ [28]. These data confirmed that MAPTAC and AM were successfully grafted into AL.

#### 3.1.2. ^1^H NMR Analysis

**Figure 3:**
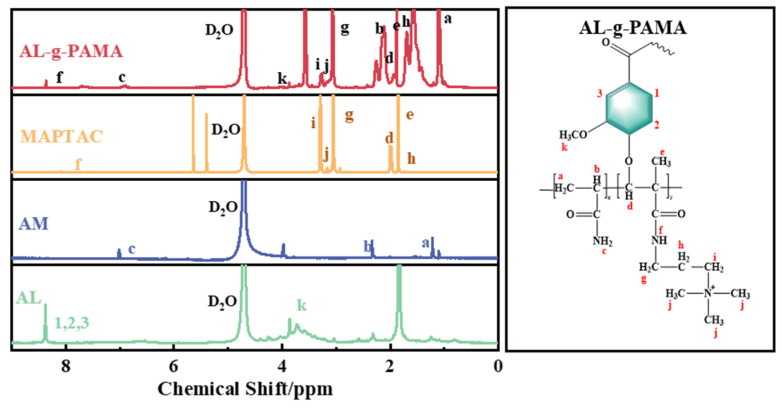
^1^H NMR spectra of AL, AM, MAPTAC, and AL-g-PAMA.

Figure 3 shows the ^1^H NMR spectra of AL, AM, MAPTAC, and AL-g-PAMA. In the ^1^H NMR spectrum of AL-g-PAMA, the chemical shift at δ = 1.11 (H_a_) ppm corresponded to the methylene group (–CH_2_) on the main chain of CPAM [29,30]. The chemical shifts at δ = 2.00 (H_d_) and 1.85 (H_e_) corresponded to the presence of hypomethyl (–CH) and methyl (–CH_3_) groups, respectively, on the main chain of MAPTAC. The chemical shifts at 3.05 (H_g_), 1.73 (H_h_), 3.29 (H_i_), and 3.17 (H_j_) ppm represented the proton peaks of the three methyl groups (–CH_2_) and three equivalent methyl groups on the quaternary ammonium moiety (–N^+^(CH_3_)_3_) in the branched chain of MAPTAC, respectively [31,32]. Additionally, AL-g-PAMA retained the benzene ring structure of AL. The chemical shift at δ = 3.90 (H_k_) ppm corresponded to the proton peak of the methoxy group (–OCH_3_), which was linked to the benzene ring in AL. Analysis of the above spectra further demonstrated that AM and MAPTAC were successfully grafted onto AL.

#### 3.1.3. XRD Analysis

**Figure 4:**
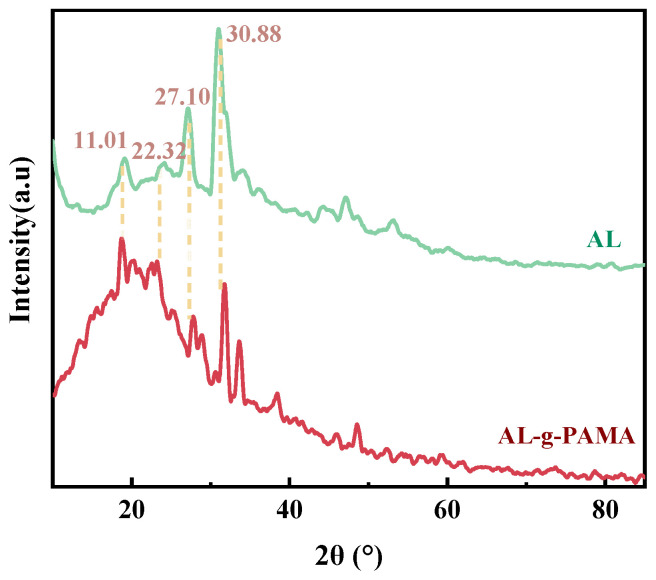
XRD patterns of AL and AL-g-PAMA.

The XRD patterns of AL and AL-g-PAMA are shown in Figure 4. The XRD pattern for AL had four peaks near 2θ = 11.01°, 22.32°, 27.10°, and 30.88°, showing a typical A-type XRD pattern [33]. Compared to the XRD pattern of AL, that of AL-g-PAMA had broad dispersed peaks, indicating that its crystallinity was lower [30,34]. This decrease in crystallinity indicated that the introduction of AM and MAPTAC led to an increase in steric hindrance within AL-g-PAMA, which weakened intra- and intermolecular hydrogen bonding, thereby resulting in a significant decrease in the overall structural order [35,36]. Therefore, AL-g-PAMA had an amorphous structure. In summary, the change in the XRD peaks of AL-g-PAMA further confirmed that AM and MAPTAC were successfully grafted onto AL.

Differences in the internal structure of flocculants inevitably cause changes in flocculation performance. The crystal structure of AL was mainly attributed to hydrogen bonding between phenolic hydroxyl groups and the ordered nature of the molecule. Poor flocculation potential in AL was mainly attributed to the lack of cationic groups that could be protonated with sludge particles and the short length of the branched chains. The grafting of AM and MAPTAC was important for reducing the degree of crystallinity of AL, which significantly improved its flocculation efficiency. This improvement likely occurred because MAPTAC provided abundant quaternary ammonium groups that enhanced electrostatic attraction between the flocculant and the kaolin particles. The long-branched structure of AM greatly strengthened bridging and netting effects. This synergistic mechanism enabled the suspended kaolin particles to combine and settle quickly during flocculation [37].

#### 3.1.4. SEM Analysis

**Figure 5:**
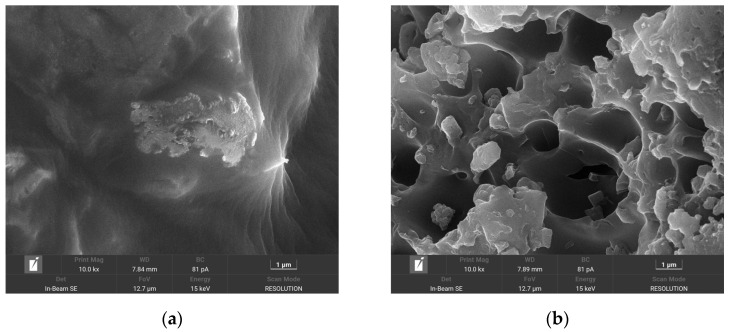
SEM images of (**a**) AL and (**b**) AL-g-PAMA.

Figure 5 shows SEM images of AL and AL-g-PAMA at 10,000× magnification. The surface of AL in Figure 5a was uniform and smooth, with relatively few folds and depressions, while the surface of AL-g-PAMA in Figure 5b was uneven, with irregular folds, many raised structures, and numerous localized hollow structures of varying sizes. This discrepancy in the morphologies likely occurred because the introduction of MAPTAC disrupted hydrogen bonding on AL, resulting in the collapse of the original crystal structure of AL and the alteration of its surface structure. This characterization also demonstrated the successful grafting of AM and MAPTAC onto AL. The SEM images demonstrated that AL-g-PAMA had a large specific surface area, increasing the possibility that it would make contact with kaolin particles. This feature somewhat enhanced the netting and sweeping effect of AL-g-PAMA on the suspended particles; therefore, the generated flocs could settle quickly [25,31].

#### 3.1.5. TG Analysis

**Figure 6:**
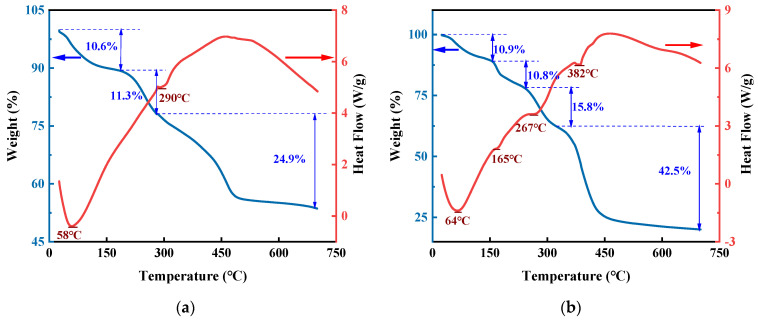
Thermal stability analysis of (**a**) AL and (**b**) AL-g-PAMA.

The stability of AL-g-PAMA was assessed through TG analysis (Figure 6). The data revealed three weight-loss stages that occurred during the thermal decomposition of AL. In the initial stage (26–187 °C, weight loss of 10.6%), bound water escaped from AL [38]; in the second stage (187–281 °C, weight loss of 11.3%), phenolic hydroxyls were oxidized and degraded, and aliphatic chains were broken and decomposed, which was accompanied by the generation of H_2_O, CO_2_, and CO; and in the third stage (281–700 °C, weight loss of 24.9%), benzene rings in AL were destroyed, and coke residue was formed [39].

In contrast to the differential TG curve of AL, the TG curve of AL-g-PAMA was divided into four weight loss stages. The first two stages were similar to those of AL. However, in the third stage (244–322 °C, weight loss of 15.8%), the amide groups in AM and MAPTAC underwent thermal decomposition and imidization, and the methyl group on the quaternary ammonium group of MAPTAC was removed [40]. When the temperature was 383 °C, there was a weak exothermic peak in the DSC curve of AL-g-PAMA. This occurred because after the introduction of AM and MAPTAC into AL, the original molecular structure of AL was destroyed, and thermal decomposition of both amide groups and the AL backbone occurred during heat treatment. These results indicated that the original thermal decomposition properties of AL were altered by the grafting of AM and MAPTAC.

### 3.2. Flocculation and Dewatering Performance

To investigate the main factors affecting flocculation performance, five representative types of AL-g-PAMA were selected for subsequent flocculation experiments, with specific details shown in Table 1.

#### 3.2.1. Effect of AL-g-PAMA Type on Flocculation Performance

**Figure 7:**
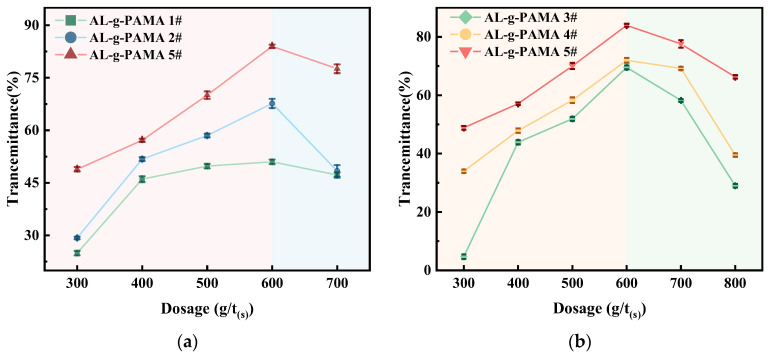
(**a**) Effect of the intrinsic viscosity of AL-g-PAMA on the transmittance of the supernatant. (**b**) Effect of AL-g-PAMA grafting efficiency on supernatant transmittance.

The intrinsic viscosity of a polymer is directly proportional to its relative molecular weight. Flocculants with higher molecular weights generally provide more active sites than those with lower molecular weights, thus forming larger flocs and exhibiting greater adsorption capacity; these features promote effective particle coagulation and settling [41]. Therefore, intrinsic viscosity is an important indicator for evaluating the flocculation performance of AL-g-PAMA [42]. As shown in Figure 7a, although the intrinsic viscosities of the three AL-g-PAMA were different, the flocculation efficiencies of the three flocculants on kaolin suspensions showed similar trends with increasing dosage. At the same dosage, the flocculation efficiency of the kaolin suspension treated with AL-g-PAMA #5 was the greatest. This likely occurred because AL-g-PAMA #5 had the highest intrinsic viscosity as well as long linear polymer chains that were able to easily form a network structure, thereby increasing the possibility of collision between AL-g-PAMA and kaolin particles [43]. Thus, the colloids formed “bridges” with each other and adsorbed more kaolin particles; these particles aggregated, flocculated, and settled to the bottom of the system.

For the AL-grafted cationic monomers in this study, the PAMA grafting efficiency was related to the amount of introduced charge. As shown in Figure 7b, the transmittance of the supernatants after AL-g-PAMA treatment with three different grafting efficiencies tended to increase and then decrease with increasing flocculant dosage. The supernatant of the kaolin suspension treated with AL-g-PAMA #5 had the highest transmittance. AL-g-PAMA #5 had the highest grafting efficiency, which indicated that this flocculant contained the greatest proportion of quaternary ammonium groups and that the surface of AL-g-PAMA #5 had more positive charges. When treating negatively charged kaolin particles, the positively charged AL-g-PAMA #5 exerted stronger electric neutralization effects and achieved better flocculation efficiency. In summary, charge neutralization and bridging played important roles in the flocculation and sedimentation process of this flocculant in kaolin suspensions.

#### 3.2.2. Effect of AL-g-PAMA #5 Dosage on Flocculation Performance

**Figure 8:**
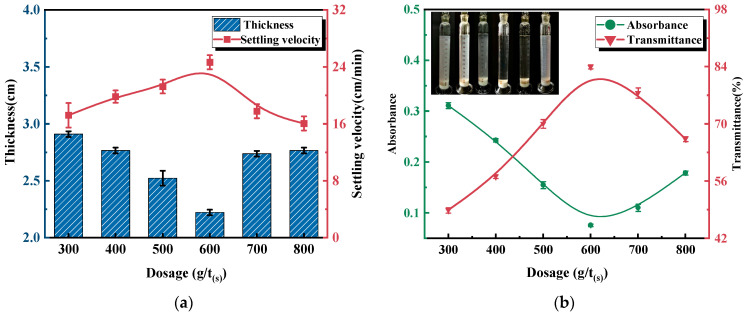
(**a**) Effect of varied t dosages on the flocculation settling velocity and thickness of the compression layer of the kaolin suspension. (**b**) Effect of different dosages on the clarity of the supernatant of the kaolin suspension.

To thoroughly investigate the flocculation performance of AL-g-PAMA, AL-g-PAMA #5 (which showed remarkable flocculation efficiency) was further considered. Figure 8a shows the effect of the AL-g-PAMA #5 dosage on the settling velocity of the kaolin suspension and the thickness of the compressed layer. The addition of AL-g-PAMA #5 improved the settling velocity of the kaolin suspension and maintained the settled film within a stable range. In contrast, an excessively fast settling velocity results in insufficient aggregation of all suspended particles, leaving some solids in the supernatant. As the dosage of AL-g-PAMA #5 increased, the settling velocity increased and then decreased, whereas the thickness of the compression layer decreased slightly and then increased. When the flocculant was present in excess, the binding sites on the surfaces of the particles became saturated. This excess flocculant did not bind effectively to the particles, creating additional voids in the composite structure. These voids resulted in instability and looseness of the resulting flocculated structure, which increased the resistance of the particles to settle in the liquid. As a result, the settling velocity of the particles decreased [24]. Simultaneously, loose flocs could lead to a thicker compression layer thickness.

Figure 8b shows the effect of different dosages of AL-g-PAMA #5 on the clarity of the kaolin suspension supernatant. This clarity increased and then decreased with increasing the flocculant dosage. The best clarity was reached when the dosage of AL-g-PAMA #5 was 600.0 g/t_(s)_ and the transmittance was 84.0%. When the dosage of AL-g-PAMA #5 exceeded 600.0 g/t_(s)_, the transmittance decreased, and the flocculation efficiency decreased. An excessive flocculant caused a decrease in the settling velocity of the particles, resulting in an increase in the content of solid particles in the supernatant, as well as the formation of larger flocs and colloidal particles. These phenomena reduced the clarity of the supernatant. The optimal settling was achieved at a dosage of 600.0 g/t_(s)_.

#### 3.2.3. Effect of AL-g-PAMA #5 Dosage on Dewatering Performance

**Figure 9:**
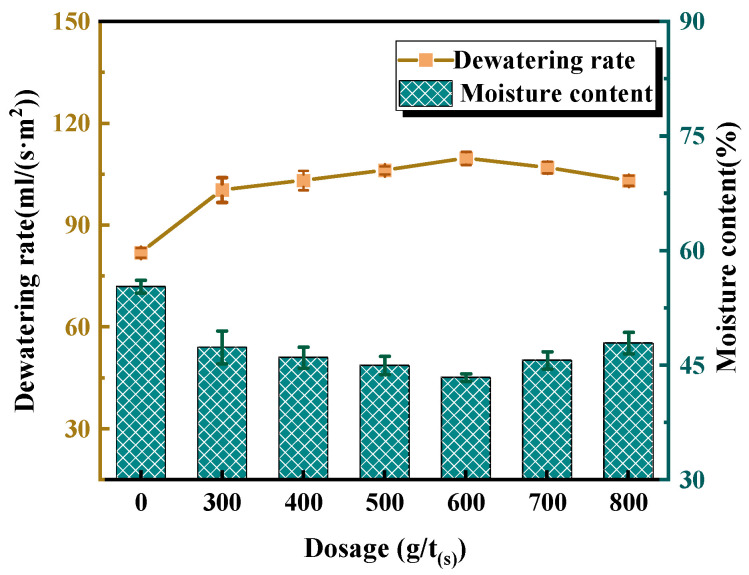
Effect of different AL-g-PAMA #5 dosages on the dewatering effect of kaolin suspensions.

Figure 9 shows the effect of varied dosages of AL-g-PAMA #5 on the dewatering of kaolin suspensions. The evaluation indices included the dewatering rate and the moisture content of the filter cake. The original kaolin filter cake had a moisture content of 55.0% and a dewatering rate of 83.6 mL/(s·m^2^) without the addition of any flocculant. When the dosage of AL-g-PAMA #5 was increased, the dewatering rate increased and then decreased, while the moisture content decreased and then increased. When the dosage of AL-g-PAMA #5 reached 600.0 g/t_(s)_, the dewatering rate reached a maximum of 110.0 mL/(s·m^2^), while the moisture content decreased to a minimum of 43.4%. Therefore, the addition of AL-g-PAMA #5 increased the dewatering rate of the suspension and reduced the moisture content of the kaolin filter cake. The addition of the flocculant enhanced interactions between kaolin particles, resulting in the aggregation into larger flocs that were easily captured and precipitated, thus increasing the dewatering rate. Moreover, the pore spaces within these aggregated flocs were reduced, making it easier for water to be discharged from the filter cake and reducing the moisture content of the cake [44,45]. When the quantity of AL-g-PAMA #5 exceeded 600.0 g/t_(s)_, the dewatering rate decreased while the moisture content increased. This might have occurred because the dosage of AL-g-PAMA #5 in the kaolin suspension was very high and the hydrogen bonding forces between particles and water molecules were strong; therefore, the solid-liquid separation was hindered, the dewatering rate slowed, and the amount of water inside the flocs increased.

### 3.3. Interaction between AL-g-PAMA and Kaolin Particles

#### 3.3.1. Zeta Potential Analysis

**Figure 10:**
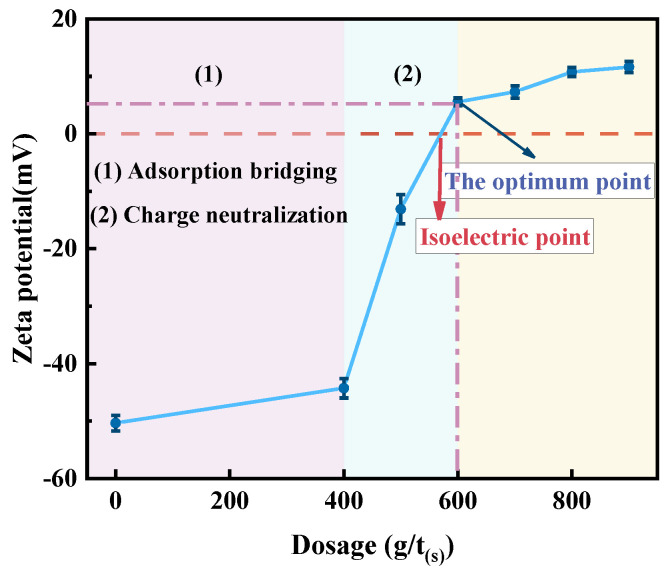
Effect of the AL-g-PAMA #5 dosage on zeta potential.

The zeta potential is commonly employed to characterize the mechanism of electrical neutralization during flocculation by analyzing changes in the electric double-layer potential of colloidal particles [46]. Figure 10 shows the variation in the zeta potential of the reaction system with changing the AL-g-PAMA #5 dosage. When the dosage of AL-g-PAMA #5 was lower than 400.0 g/t_(s)_, the zeta potential of the supernatant increased slowly with increasing the AL-g-PAMA #5 dosage. In this case, the flocculation mechanism of AL-g-PAMA #5 was mainly driven by the bridging and net trapping effects. When the dosage was 400.0–600.0 g/t_(s)_, the zeta potential increased rapidly and shifted from a negative to a positive potential. In this case, the flocculation mechanism of AL-g-PAMA #5 was mainly driven by charge neutralization. The surface charge of the negatively charged suspended kaolin particles was rapidly neutralized, and the particles were destabilized, such that the supernatant was positively charged [47]. When the dosage of AL-g-PAMA #5 exceeded 600.0 g/t_(s)_, the increase in zeta potential slowed. Excess flocculant remained on the surface of the kaolin particles to form a covering layer that prevented direct interactions between the charges in the solution and the particles and slowed the increase in the zeta potential. In this case, the flocculation efficiency deteriorated, indicating that “restabilization” occurred, which was detrimental to the flocculation performance [48].

#### 3.3.2. FBRM Results

**Figure 11:**
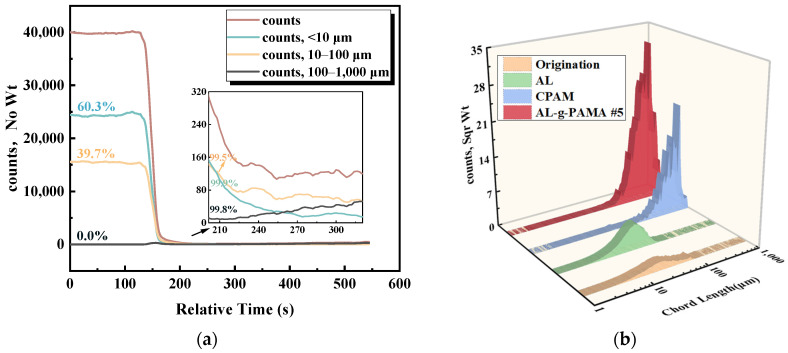
FBRM analysis curves. (**a**) Variation in the number of particles of different sizes after treatment with AL-g-PAMA #5. (**b**) Particle size distribution of kaolin particles using different types of flocculants.

Figure 11a shows the distribution of the number of particles of different sizes with time during flocculation after the addition of AL-g-PAMA #5. The number of particles in the kaolin suspension system before the addition of flocculant was approximately 40,000. A total of 60.3% of the particles had a size less than 10 μm; 39.7% consisted of 10–100 μm particles; and less than 0.01% consisted of larger than 100 μm particles. After the system was homogeneously stirred for 2 min, the flocculant was added. Then, the number of particles in the system rapidly decreased, with the number of particles smaller than 10 μm decreasing by 99.9% and the number of particles in the size range of 10–100 μm decreasing by 99.5%; however, the number of particles in the size range of 100–1000 μm increased. The high positive charge and long-chain structure of AL-g-PAMA #5 led to the continuous aggregation of fine particles smaller than 100 µm. The formation of larger flocs was beneficial for improving the settling efficiency [49].

Different types of flocculants have different effects on supernatant clarity [24]. Therefore, the dynamic distribution of particles during the flocculation of kaolin suspensions by AL-g-PAMA #5 was investigated using FBRM. Figure 11b shows the dynamics of the number of kaolin particles with time after the addition of different types of flocculants. The chord length of the original sample was 27 μm. After the addition of AL, the chord length distribution of the kaolin particles was similar to that of the original sample, albeit with more dispersion, and the chord lengths of most of the particles did not change. This result demonstrated that lignin did not enable flocculation. The size of the flocs increased significantly with the addition of CPAM and AL-g-PAMA #5. CPAM and AL-g-PAMA #5 had similar chord length distributions. The chord lengths of most of the particles with CPAM were between 570 and 630 μm, while the chord lengths of the particles with AL-g-PAMA #5 mainly ranged from 480 to 550 μm. Moreover, AL-g-PAMA #5 contained more particles than CPAM. These results confirmed that flocculation efficiency improved after treatment with AL-g-PAMA #5 [50].

The AL-g-PAMA #5 and commercial flocculant (CPAM) results for flocculation and dewatering of the kaolin suspension at a dosage of 600.0 g/t_(s)_ are shown in Table 2. Compared with the results for CPAM, the kaolin suspension treated with AL-g-PAMA #5 had a moderate settling velocity, a clear supernatant, a thinner compression layer, and a lower moisture content in the filter cake. Considering the FBRM analysis curve shown in Figure 11, the kaolin flocs treated with CPAM were larger, and their settling velocity increased. The CPAM-treated flocs were loose, resulting in lower floc compressibility and a thicker compression layer. The oversized flocs contained more water; therefore, the moisture content of the filter cake increased after dewatering. The kaolin particles treated with AL-g-PAMA #5 were tightly aligned, forming flocs with small pores and dense structures. The flocs of kaolin particles treated with AL-g-PAMA #5 contained less water, and the dewatering efficiency improved [51]. Currently, mine wastewater treatment is increasingly being performed via integrated precipitation and dewatering, and the excellent dewatering effect of AL-g-PAMA is conducive to improving the solid-liquid separation of suspensions in these systems. In addition, CPAM is biodegradable and toxic, and the use of CPAM carries the risk of secondary pollution. Therefore, AL-g-PAMA can replace CPAM as an environmentally friendly and efficient flocculant for the solid-liquid separation of kaolin suspensions.

#### 3.3.3. Flocculation Mechanism of AL-g-PAMA

There are three main mechanisms involved in the proposed flocculation process: electroneutralization, adsorption and bridging, and netting and sweeping [52]. In general, the internal structure of the flocculant has a large influence on the flocculation and dewatering performance of kaolin [29]. In this study, the successful synthesis of AM and MAPTAC on the AL backbone was demonstrated by a series of characterization methods. AM was introduced to increase the molecular weight of AL and strengthen adsorption-driven bridging during the flocculation process. MAPTAC was introduced to increase the number of positive charges on AL and enhance adsorption and electroneutralization during flocculation. MAPTAC, which has a long molecular chain, large side groups, and a large molecular volume, strengthened bridging and net trapping during flocculation [52]. Moreover, SEM analysis (see Figure 5) revealed that many pores of different sizes were distributed on the surface of AL-g-PAMA, indicating that its specific surface area increased. Thus, the bridging and netting effects were enhanced. Subsequently, these fine pores served as drainage channels, greatly improving the filterability and compressibility of the sludge.

In addition, the type and dosage of flocculants have important effects on the flocculation mechanism. In this study, the transmittance (see Figure 7) and zeta potential (see Figure 10) of the supernatants from different AL-g-PAMA flocculation treatments were analyzed to identify the mechanism underlying the corresponding flocculation processes. When the AL-g-PAMA dosage was in a range of 0–400.0 g/t_(s)_, the zeta potential increased slowly, and the improvement in the supernatant clarity was attributed to the adsorption bridging and net trapping effects of AL-g-PAMA. Under optimal conditions, the dominant flocculation mechanism was electroneutralization. Considering the effect of the AL-g-PAMA #5 dosage on the dewatering performance (see Figure 9) and the FBRM curve (see Figure 11), the chord lengths of the AL-g-PAMA #5 particles increased from 27 μm to approximately 510 μm. Large kaolin flocs formed a denser filter cake with a better dewatering performance. The floc size plays a crucial role in kaolin dewatering, which is associated with electroneutralization, as well as the net trapping and sweeping effects of AL-g-PAMA [53].

**Figure 12:**
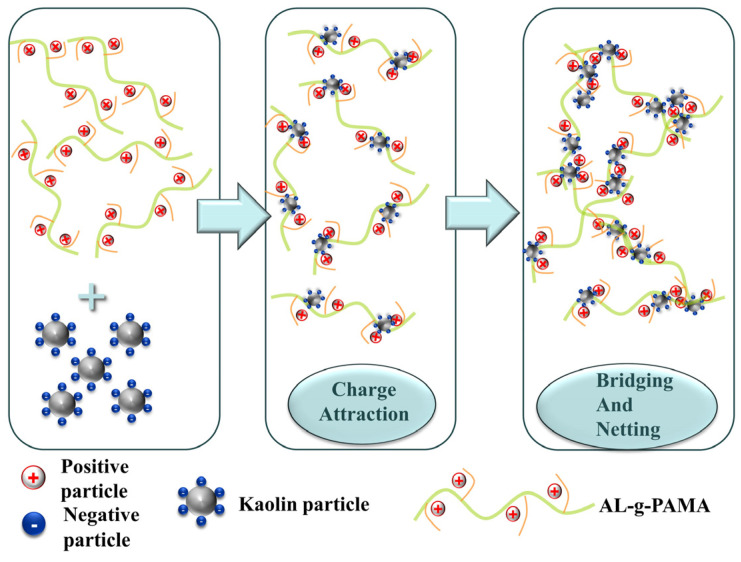
AL-g-PAMA flocculation mechanism.

Based on this discussion, the flocculation mechanism of AL-g-PAMA in kaolin suspensions is schematically shown in Figure 12. First, the positively charged quaternary ammonium and amino groups in AL-g-PAMA enhance charge adsorption during flocculation. At this stage, negatively charged kaolin particles are bound to the surface of AL-g-PAMA and subsequently aggregate by collision under electrostatic attraction. Then, due to the high ductility of AL-g-PAMA, the unstable colloidal particles aggregate into large and dense flocs by bridging and reticulation. Eventually, the flocs settle to the bottom of the settling tube. Due to these mechanisms, AL-g-PAMA showed efficient flocculation and dewatering effects.

## 4. Conclusions

In this study, AM and MAPTAC were investigated as graft monomers to modify AL. A novel cationic flocculant (AL-g-PAMA) was synthesized by thermally initiated copolymerization. The FTIR, ^1^H NMR, XRD, TG, and SEM results confirmed the successful synthesis of AL-g-PAMA. The SEM results showed that AL-g-PAMA had a porous and uneven surface structure.

The addition of AL-g-PAMA significantly improved the flocculation, sedimentation, and dewatering of the kaolin suspension. When the dosage of AL-g-PAMA #5 was 600.0 g/t_(s)_, the thickness of the compressed layer was 2.2 cm, the settling velocity was 24.1 cm/min, and the transmittance of the supernatant was 84.0%. These values corresponded to the optimal conditions for flocculation of the kaolin suspension. The flocculation efficiency of AL-g-PAMA #5 was equivalent to that of CPAM. Furthermore, vacuum filtration was conducted at this dosage, reducing the moisture content of the resulting filter cake by 11.6%. The moisture content of the kaolin filter cake treated with AL-g-PAMA was 4.3% lower than that of the kaolin filter cake treated with CPAM.

AL-g-PAMA exhibited excellent flocculation performance on suspended particles due to the strong net trapping and electroneutralization effects of the grafted AM and cationic MAPTAC.

Organic flocculants, such as CPAM, exhibit problems, such as high toxicity and resistance to degradation. In contrast, AL-g-PAMA is a highly efficient, low-consumption, and environmentally friendly flocculant that provides a novel approach to the treatment of kaolin suspensions.

## Figures and Tables

**Table 1:** Details of the experimental flocculants.

Flocculant	Weight of AL (g)	Weight of AM (g)	Volume of MAPTAC (mL)	Weight of KPS (g)	Volume of the Solution (mL)	Intrinsic Viscosity (dL/g)	Grafting Efficiency of PAMA (%)
AL-g-PAMA #1	8.7	21.8	12.6	2.0	100	3.4	77.1
AL-g-PAMA #2	8.7	21.8	12.6	3.0	100	4.7	79.8
AL-g-PAMA #3	11.2	36.5	8.1	4.0	100	5.3	67.5
AL-g-PAMA #4	8.7	21.8	12.6	5.0	100	6.6	73.8
AL-g-PAMA #5	8.7	21.8	12.6	4.0	100	6.4	87.7
CPAM	-	-	-	-	-	7.6	-

CPAM: commercial polyacrylamide.

**Table 2:** Flocculation and dewatering performance of different flocculants.

Flocculant Type	Transmittance (%)	Settling Velocity (cm/min)	Thickness of the Compressed Layer (cm)	Filter Cake Moisture Content (%)
AL-g-PAMA #5	84.0 ± 0.5	24.1 ± 1.0	2.2	43.4 ± 0.5
CPAM	84.2 ± 1.1	31.0 ± 1.7	2.8	47.7 ± 1.1

## Data Availability

Data are contained within the article.
